# Lactylated Proteomic Analysis Reveals Functional Implications of Lysine Lactylation In Asthenozoospermia

**DOI:** 10.1016/j.mcpro.2025.101439

**Published:** 2025-11-04

**Authors:** Minghao Yan, Haixia Tu, Shanshan Tang, Zhengxiao Gai, Qiancheng Shi, Yueshuai Guo, Kai Wang, Xianlin Xu, Xuejiang Guo, Yan Li

**Affiliations:** 1 Department of Clinical Laboratory, Sir Run Run Hospital, Nanjing Medical University, Nanjing, China; 2 Department of Urology, Sir Run Run Hospital, Nanjing Medical University, Nanjing, Jiangsu Province, China; 3 State Key Laboratory of Reproductive Medicine and Offspring Health, Department of Histology and Embryology, Nanjing Medical University, Nanjing, China

**Keywords:** lactylation, male infertility, asthenozoospermia, PGK2, proteomics

## Abstract

The mechanism underlying asthenozoospermia in male infertility has been a prominent topic in reproductive medicine research. Human sperm function is modified by various protein post-translational modifications (PTMs). Among these, lactylation modification, a relatively novel PTM, has not been previously reported in the context of the male reproductive system. Comparative analyses between asthenozoospermic and normal sperm have revealed a significant down-regulation in the level of lysine lactylation (Kla) in proteins from asthenozoospermic sperm. Based on proteomic studies of protein Kla, 220 lactylated proteins were identified in sperm. Bioinformatics results showed that these lactylated proteins were highly enriched in the glycolytic pathway. Phosphoglycerate kinase 2 (PGK2), a key glycolytic enzyme and testis-specific protein, has been found to have 10 lactylated sites (K6, K11, K31, K41, K141, K192, K220, K272, K322, and K353). In asthenozoospermic sperm, both the lactylation level of PGK2 and its enzyme activity were reduced, while exogenous supplementation with PGK2 downstream products ameliorated sperm motility dysfunction. Mutation experiments at the K220 site confirmed that PGK2 (K220) lactylation affects glycolysis by regulating its enzyme activity. This study provides the first evidence of the regulatory role of proteins lactylation in sperm function.

## Highlights

• Proteomic analysis of human sperm identified 220 lysine lactylated proteins.
• Protein lactylation is downregulated in asthenozoospermic sperm.
• The lactylation of PGK2 (K220) regulates its enzyme activity and ATP production.
• Downstream products of PGK2 rescue motility defect in asthenozoospermia.

## Graphical Abstract

**Figure:**
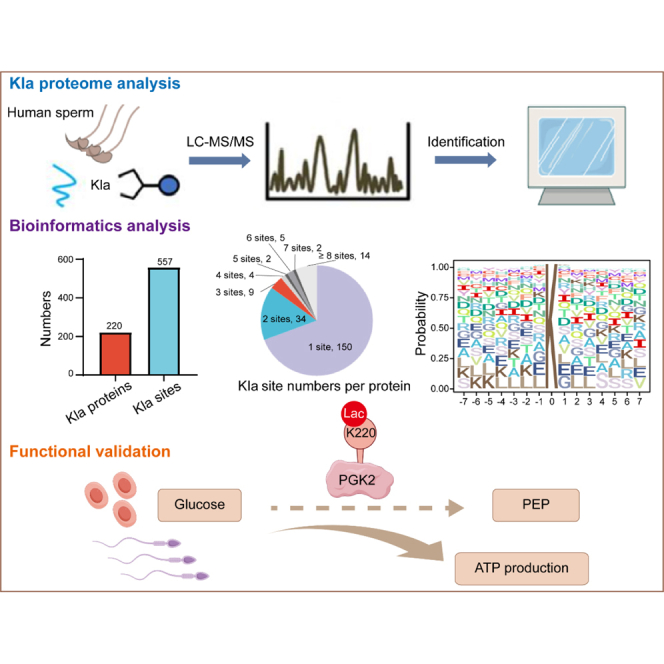

## In Brief

The role of protein lactylation in sperm function remains unclear. Our lysine lactylation proteome analysis of human sperm identified 220 lactylated proteins enriched in glycolytic pathway and sperm motility. The lactylation level of PGK2, a key enzyme in glycolysis, were significantly reduced in asthenozoospermia. Supplementation of its downstream products partially restored sperm motility. PGK2 (K220) lactylation is important for its enzymatic activity in glycolysis. Abnormal protein lactylation is an important mechanism in asthenozoospermia, providing a new direction for its treatment.

The infertility rate is increasing annually, with data from the World Health Organization (WHO) indicating that the global infertility rate has reached 17.5%, with male factors contributing to approximately 50% of cases (1, 2). Clinically, this is manifested by reductions in sperm count, decreased motility, and abnormalities in sperm morphology (2). Asthenozoospermia (AZS) is a prevalent cause of male infertility, about 81% of male infertility is caused by AZS, which is characterized by sperm motility below the WHO standard, specifically when the percentage of sperm progressive motility is less than 32% (3, 4, 5). Worldwide, there is a decline in male semen quality, and the prevalence of AZS has risen significantly, highlighting its importance as a public health concern in reproductive health (6, 7). Consequently, understanding the mechanisms underlying AZS has become a high priority in the field of reproductive medicine.

Sperm is one of the most differentiated cell types in the male body. During sperm formation, its chromatin is progressively replaced from the original histones by protamine, eventually reaching about 85% protamine within the chromatin (8). Mature sperm lack transcriptional competence, and their cytoplasmic ribosomes are translationally inactive (9). Consequently, the regulation of sperm function predominantly occurs at the protein level, and protein post-translational modifications (PTMs) play important roles in sperm function (10, 11, 12). It has been shown that the human sperm flagellum is highly dependent on glycolysis for ATP production and produces large amounts of lactate (13). The recent groundbreaking discovery that lactate can contribute to the formation of histone lactylation has unveiled a novel protein modification by liquid chromatography-tandem mass spectrometry (LC-MS/MS) (14, 15). Subsequent studies showed that lysine lactylation (Kla) is evolutionarily conserved and prevalent across a range of organisms, from unicellular entities to humans (16, 17, 18, 19). Protein Kla has been found to function in various human diseases, such as tumors, inflammation, and neurological disorders (20, 21). For example, lactate promotes cell proliferation and migration via histone H3K18 lactylation in pancreatic ductal adenocarcinoma (22). And H4K12 lactylation could activate the transcription of genes essential for ATC anaplastic thyroid cancer proliferation (23). Nevertheless, as a novel addition to the repertoire of PTMs, the role of lactylation in the regulation of the male reproductive system, particularly sperm function, remains largely unexplored and warrants investigation.

In this study, we found that sperm proteins are lactylated. Our Kla proteome analysis identified a total of 220 lactylated proteins in human sperm. Bioinformatics analysis indicated that these lactylated proteins are enriched in biological processes related to the glycolytic pathway and sperm motility. Notably, lactylation of PGK2, a key enzyme in the glycolytic pathway, is significantly down-regulated in AZS sperm. Upregulation of PGK2 (K220) lactylation or exogenous supplementation of the downstream product of PGK2 improved sperm glycolysis and motility. The enzyme activity is regulated by lactylation of PGK2 (K220). The abnormal sperm protein lactylation is an important mechanism in AZS, which provides a new approach to the treatment of AZS.

## Experimental Procedures

### Experimental Design and Statistical Rationale

The methods and experimental protocols for all human subjects were approved by the Ethics Committee of the Sir Run Run Hospital of Nanjing Medical University (2022-SR-043). The study followed the principles of the Declaration of Helsinki, and written informed consent was obtained from the patients. Three biological replicates were established, each comprising sperm samples from 20 healthy male donors. Lactylome profiling was performed using LC-MS/MS in data-independent acquisition (DIA) mode. Data are expressed as mean ± standard deviation (SD). Significance analysis between two groups was determined by Student’s independent *t* test. Significance of differences between multiple groups was determined by one-way ANOVA followed by Dunnett's multiple comparisons test. *p-*value <0.05 was considered significant. All experiments were repeated at least three times.

### Sample Collection

Semen samples were collected from patients with asthenozoospermia (AZS) and healthy individuals, and after collection in sterile containers, the samples were liquefied at 37 °C for 30 min and assayed for sperm motility using a computer-assisted semen analyzer (CASA, IVOS, Hamilton Thorne). AZS samples were defined as sperm progressive motility percentage (PR) < 32% or total sperm motility (PR + NP) < 40% (3).

Sperm samples were obtained by Percoll (GE health) density gradient centrifugation. The 60% Percoll solution was added to the bottom of a 15 ml centrifuge tube (50% Percoll solution was used for AZS specimens), followed by slow addition of an equal amount of liquefied semen sample to the top of the Percoll solution and centrifugation at 300*g* for 5 min at room temperature. The supernatant was discarded, and the sperm pellet was washed twice by adding prewarmed HTF medium (Irvine Scientific). Finally, the sperm precipitate obtained was resuspended in HTF medium for subsequent experiments.

### Proteomic Analysis of Lysine Lactylation (Kla) of sperm proteins

Three normal semen specimens were collected, and sperm were obtained by 60% Percoll solution. Sperm were lysed by sonication in a buffer containing 8 M Urea, 75 mM NaCl, 50 mM Tris (pH 8.2), 10 mM DTT, protease/phosphatase inhibitors, 3 μM TSA, and 50 mM NAM. The lysate was incubated on ice for 1 h, centrifuged at 30,000*g* for 60 min at 4 °C, and the supernatant was collected and quantified using the Bradford assay. Proteins were reduced, alkylated, trypsinized, and desalted using an OASIS HLB Vac cartridge. Lactylated peptides were enriched with anti-L-Lactyl Lysine antibody-conjugated agarose beads (PTM Bio), eluted, dried, desalted, and analyzed by LC-MS/MS using an Orbitrap Fusion Lumos mass spectrometer.

Enriched lactylated peptides were dissolved in 0.1% formic acid (FA), and analyzed using an Orbitrap Fusion Lumos mass spectrometry system (ThermoFisher) coupled with the Easy-nLC 1200 (ThermoFisher). Solvent A consisted of 0.1% FA, while solvent B comprised 80% acetonitrile (ACN) and 0.1% FA. The peptides were separated using an analytical column (75 μm × 150 mm, 1.7 μm, CoAnn Technologies) with a 60 min gradient (3%–5% B for 5 s, 5%–15% B for 23 min and 55 s, 15%–28% B for 21 min, 28%–38% B for 7 min and 30 s, 38%–100% B for 5 s, and 100% B for 7 min and 25 s).

DIA was performed with the following parameters. MS1 scans were acquired at a resolution of 120 k, covering a mass range of 350 to 1500 m/z, with an AGC target of 1E6 and a maximum injection time of 50 ms. For MS/MS analysis, higher-energy collisional dissociation (HCD) was employed with a normalized collision energy of 30%, a resolution at 30 k, dynamic maximum injection time, and an AGC target of 5E5. Isolation windows were set based on the signal distribution of tryptic peptides in this experiment. These included 50 Da for precursor ions in the 350 to 450 m/z range, 15 Da for those in the 450 to 960 m/z range, and 100 Da for those in the 960 to 1500 m/z range.

The raw MS/MS data were analyzed with Spectronaut (Biognosys, v19.0) using directDIA. Data was searched against the *Homo sapiens* database from the Universal Protein Resource (UniProt, 2024.07; 20,420 entries). Trypsin/P was specified with two missing cleavages. Carbamidomethylation was set as a fixed modification for cysteine, and lactylation on lysine and oxidation on methionine were set as variable modifications. The precursor and fragment tolerance were set as dynamic, which means Spectronaut calculated the ideal mass tolerances for data extraction and scoring based on its extensive mass calibration. The minimal peptide length was set as seven. The FDR thresholds for modification sites, peptides, and proteins were all set at 1%. Kla sites with localization probability <0.75 were excluded.

### Bioinformatics Analysis

Frequency and enrichment of amino acid residues surrounding the Kla sites were performed by IceLogo. Subcellular localization of lactylated proteins was predicted by WoLF PSORT. Gene Ontology (GO) and Kyoto Encyclopedia of Genes and Genomes (KEGG) annotations, as well as protein visualization were performed by ClusterProfiler with an adjusted *p*-value of less than 0.05 as the threshold.

### Immunofluorescence Staining

The obtained human sperm was spread on a slide and air-dried at room temperature. After fixation with 4% paraformaldehyde for 15 min, 0.1% Triton X-100 was added dropwise for 10 min for permeabilization. Subsequently, 5% BSA was added to block for 1 h. The samples were incubated with anti-pan-Kla (PTM Bio) and anti-Ac-tubulin (Sigma) at 4 °C overnight. The next day, add the corresponding fluorescent secondary antibodies (anti-rabbit IgG Alexa Fluor 488 and anti-mouse IgG Alexa Fluor 594) and incubate for 1 h. Add Hoechst and incubate for 20 min. The slices were sealed with glycerol and placed under a fluorescence confocal microscope (TCS SP8X, Leica) for observation and photography.

### Immunoprecipitation (IP)

To the obtained sperm/specimen, IP lysis solution (Thermo) was added, lysed on ice for 10 min and centrifuged to collect the supernatant. 10 μg/mg PGK2 antibody (Proteintech) and IgG antibody (Beyotime) solutions were added separately and incubated overnight at 4 °C in a turnover mixer to obtain antigen-antibody complexes. The magnetic beads (MCE) were thoroughly mixed, and 30 μl were taken and washed with washing buffer. The antigen-antibody complex was added to the magnetic beads and incubated for 2 h at 4 °C in a Flip Mixer. After washing 4 times, add 1 × loading buffer and incubate for 5 min at 95 °C. Separate the beads and collect the supernatant to obtain the sample.

### Sperm Culture and Treatment

The obtained normal human sperm specimens were resuspended in HTF medium and incubated for 1 or 2 h by adding 5, 10, 20 mM lactate solution (MCE) and 5, 10, 50 nM rotenone solution (Sigma), respectively. Add 5, 10, 20 mM oxamate solution (MCE) and incubate for 0.5 or 1 h. In IP *et al*. experiments, 20 mM lactate and 50 nM rotenone were added and incubated for 2 h and 20 mM oxamate for 1 h, respectively. In rescue experiments, 20 mM oxamate was incubated for 1 h, while ATP (Beyotime) and 3-phosphoglycerate (3-PG, Sigma) were added for 1 h.

### PGK2 Enzyme Activity Assay

Extraction solution was added to the obtained sperm/cells, and the cells were broken by ultrasonication in an ice bath (300 w, 3 s on/7 s off). After centrifugation at 10,000*g* for 10 min, the supernatant was carefully collected. Then, 900 μl working solution and 100 μl sample were added to the cuvette, and the absorbance value (A1) was measured immediately at 340 nm, followed by incubation at 37 °C for 5 min and measurement of absorbance value (A2). The concentration of the sample (mg/ml) was also determined using the BCA kit (Vazyme). Finally, PGK2 enzyme activity was calculated according to the instructions (Solarbio).

## ATP Detection

Add lysate to the obtained sperm/cell samples, lysing the cells fully by repeated blowing using a pipette, lysing on ice for 10 min and then centrifuging and carefully collecting the supernatant. Pre-add 100 μl of ATP assay working solution (MCE) to all assay wells and incubate at room temperature for 5 min to consume background ATP. Add 10 μl of sample or standard into the assay wells and shake to mix well. Immediately determine the relative light units (RLU) using a multifunctional microplate reader (CYT4000, SPARK) and calculate the concentration of ATP in the sample after drawing the standard curve.

### Phosphoenolpyruvate (PEP) Detection

Lysis solution was added to the obtained sperm/cell samples, lysed on ice for 10 min and centrifuged, and the supernatant was carefully collected. Prepare Amplex Red reaction working solution according to the kit instructions (Beyotime). Add 20 μl of sample or standard to the assay wells, followed by 80 μl of Amplex Red reaction solution to each well. Mix and react for 30 min at 37 °C, protected from light. The OD values were detected using a multifunctional microplate reader at 570 nm, and the standard curve was plotted to calculate the PEP content.

### Cell Transfection

The 293T cells were inoculated into 6-cm cell culture dishes, and the cells were transfected when the cell density reached 70%. The DNA plasmid (8 μg/well) and Lipofectamine 3000 (Thermo) were diluted separately with opti-MEM (Thermo) and left to stand at room temperature for 5 min, and then mixed. The mixture was added drop-by-drop to the cell culture wells after 15 min, replaced with complete medium after 6 h, and assayed 48 h after transfection.

### Western Blot

Protein lysis solution (Beyotime) containing protease inhibitors (MCE) was added to the obtained sperm samples, placed on ice for 20 min, centrifuged and the supernatant was collected to obtain sperm proteins. Protein concentration was measured using the BCA protein analysis kit. An equal amount of protein was then added to a 12% SDS-PAGE gel for electrophoresis and transferred to the 0.22 μm PVDF membrane (Borel). Locked in 5% milk for 1 h at room temperature. Add anti-Pan-Kla, anti-PGK2 antibody and incubate at 4 °C overnight. The next day, the corresponding HRP-conjugated secondary antibodies were incubated for 1 h at room temperature. The protein bands were then visualized using an ECL chemiluminescence kit (Vazyme), and the images were saved for analysis.

## Results

### The Levels of Sperm Protein Lactylation are Reduced in AZS

**Fig. 1:**
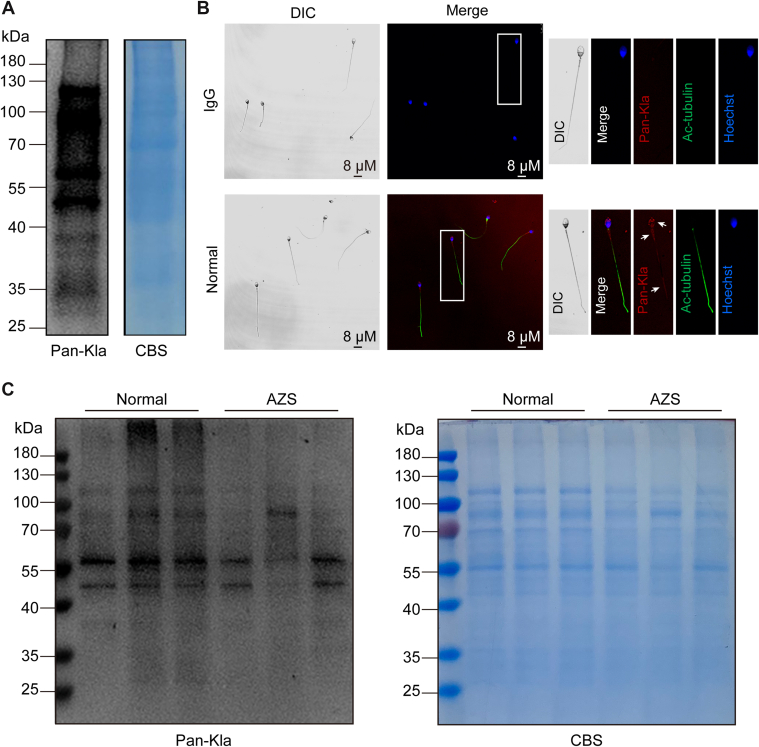
**The expression of lactylated proteins decreased in AZS.***A*, lactylation in human sperm proteins was detected by Western blot. *B*, the localization of lactylated protein in human sperm was detected by immunofluorescence staining. *C*, Western blot and Coomassie brilliant blue staining (CBS) were used to observe the expression of lactylated proteins in AZS.

Clinical specimens of normal semen were collected, and sperm were sorted using Percoll density gradient centrifugation. Western blot analysis revealed that lysine lactylated proteins were distributed across various molecular weights, suggesting the presence of multiple lysine lactylated proteins in normal human sperm (Fig. 1*A*). Immunofluorescence staining further demonstrated that these lactylated proteins were distributed on the sperm flagellum, acrosome and nucleus (Fig. 1*B*). Analysis of clinical AZS sperm samples indicated that the level of sperm protein Kla in AZS was significantly lower than in normal controls (Fig. 1*C*). This finding suggests that abnormal Kla of proteins may be an important mechanism underlying sperm motility impairment in AZS.

### Sperm Motility and Protein Lactylation are Regulated by Lactate

**Fig. 2:**
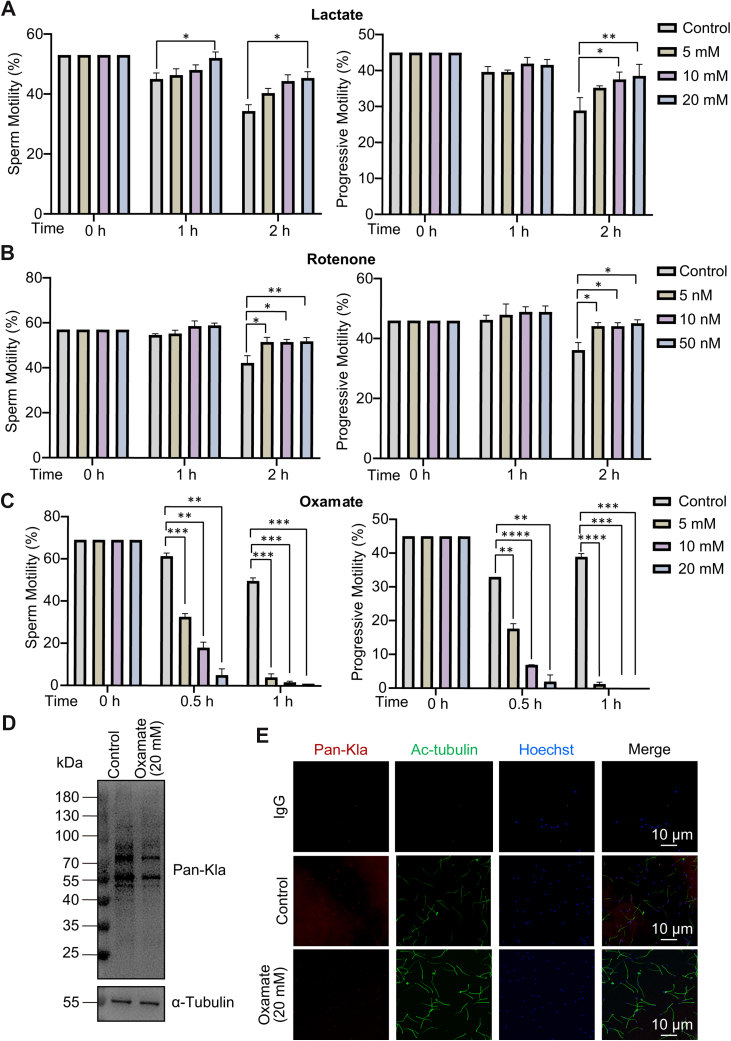
**Lactate has a regulatory effect on sperm motility.** Different concentrations of exogenous (*A*) sodium lactate (0, 5, 10, 20 mM), (*B*) rotenone (0, 5, 10, 50 nM), and (*C*) oxamate (0, 5, 10, 20 mM) were added to normal liquefied human semen, and sperm motility and progressive motility were measured by CASA analyzer. *D* and *E*: The effect of oxamate on sperm protein lactylation was detected by Western blot and immunofluorescence staining. All values are mean ± SD, ∗*p* < 0.05, ∗∗*p* < 0.01, ∗∗∗*p* < 0.001, ∗∗∗∗*p* < 0.0001.

To clarify the effect of lactylation on sperm motility, exogenous sodium lactate was added to normal semen specimens to elevate the lactate level, and the results showed that lactate enhanced sperm motility and progressive motility (Fig. 2*A*). Rotenone is an inhibitor of mitochondrial respiratory chain complex I, which drives cellular glycolysis and elevates intracellular protein lactylation modifications. The addition of rotenone also significantly elevated sperm motility and progressive motility (Fig. 2*B*). Oxamate inhibits lactate production by modulating lactate dehydrogenase activity to reduce the level of protein lactylation modification. The addition of oxamate to normal semen reduced both sperm motility and progressive motility in a concentration-dependent manner (Fig. 2*C*). To evaluate the protein lactylation changes, we performed Western blot and immunofluorescence analysis. The results showed that oxamate inhibited sperm protein lactylation (Fig. 2*D*), and protein lactylation in sperm flagellum was significantly reduced (Fig. 2*E*). The above results suggested that the lactate elevated level of protein lactylation in the sperm flagellum and enhanced sperm motility.

### Proteomic Profiling Revealed Diverse Lysine Lactylated Proteins in Human Sperm

**Fig. 3:**
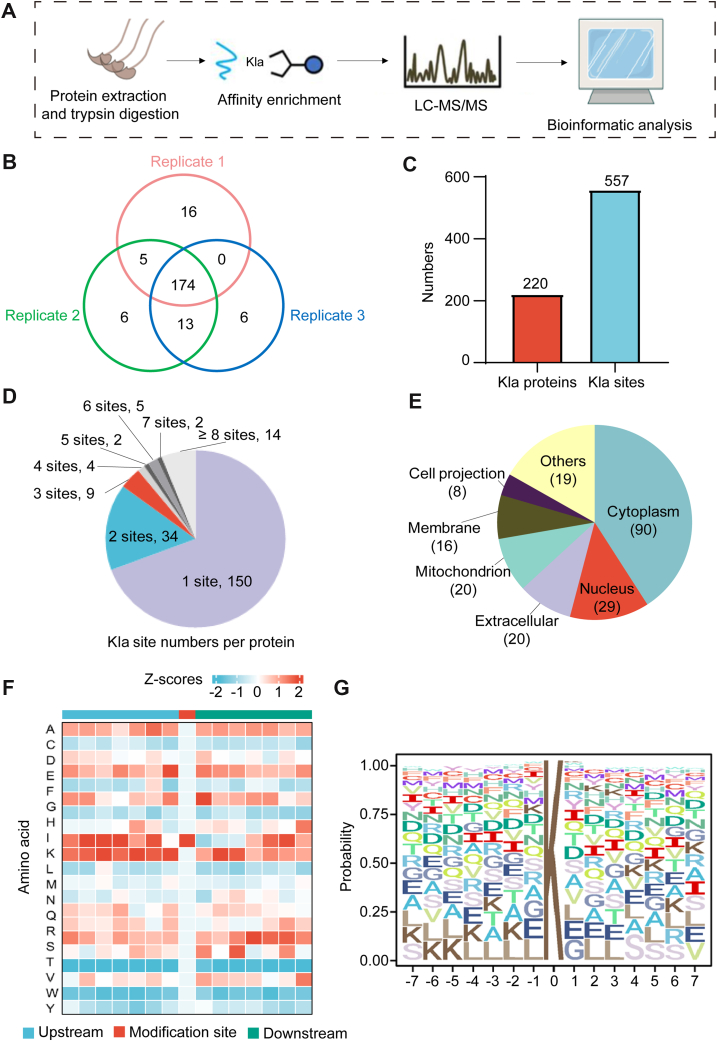
**Lactate has a regulatory effect on sperm motility.***A*, schematic representation of sperm lactylation-modified proteomics. *B*, Venn diagram shows three cases of sperm lactylated proteins. *C*, numbers of sperm lactylated proteins and lactylated sites. *D*, number of sites per lactylated protein. *E*, cellular localization of sperm lactylated proteins. The distribution of amino acid residues of sperm lactylated proteins is plotted by (*F*) heat maps and (*G*) Motif-X.

To investigate which sperm proteins are lactylated, we performed Kla proteomics analysis of human sperm (Fig. 3, *A* and *B*). The analysis revealed that 220 proteins in normal sperm exhibited lactylation, encompassing a total of 557 lactylation sites (Fig. 3*C*). Of these, 150 lactylated proteins had only one lactylation site, and 14 lactylated proteins had more than eight lactylation sites (Fig. 3*D*). More than half (119/220, 54.1%) of the lactylated proteins were localized within the cytoplasm and nucleus, while the remainder were mainly distributed in the mitochondria (20/220, 9.1%), extracellular region (20/220, 9.1%), and membrane (16/220, 7.3%) (Fig. 3*E*). Furthermore, Motif-X program was used to analyze the distribution of amino acids around the lactylated sites, and we observed that K and L were the two most enriched amino acids (Fig. 3, *F* and *G*).

### Sperm Lactylated Proteins are Enriched in the Glycolytic Pathway

**Fig. 4:**
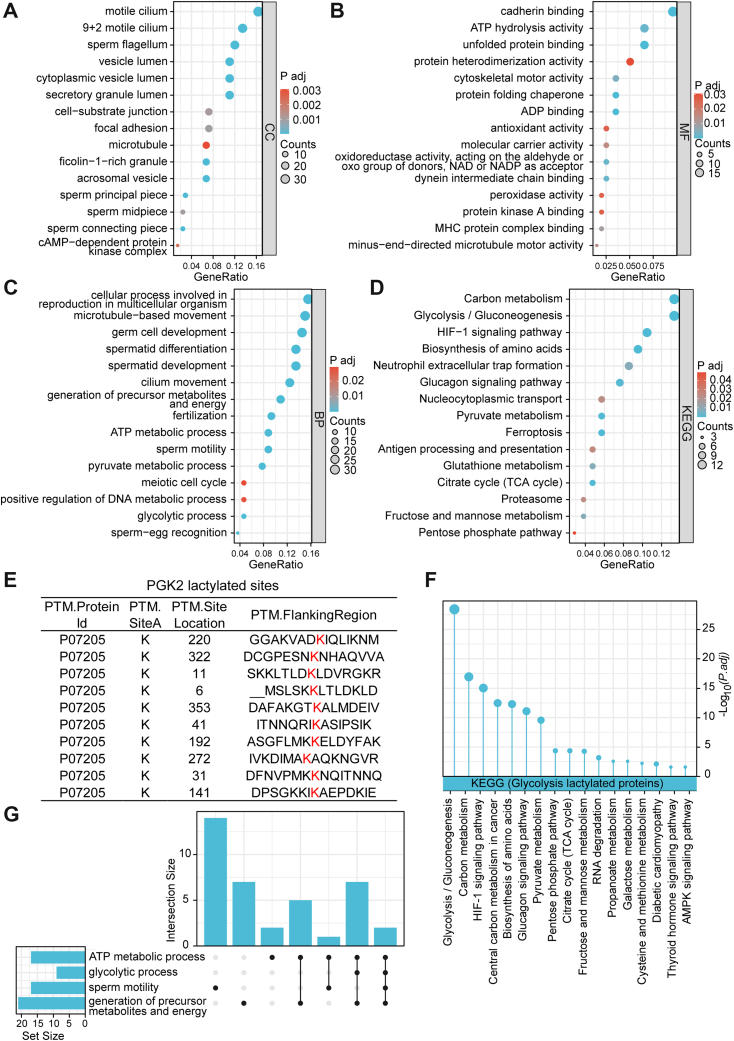
**Bioinformatics analysis of sperm lactylated proteins.** Gene Ontology annotated sperm lactylated proteins. Enriched terms for (*A*) cellular components, (*B*) molecular function, and (*C*) biological processes and cellular components are shown. *D*, KEGG pathway enrichment analysis of sperm lactylated proteins. *E*, PGK2 lactylated sites. *F*, KEGG pathway enrichment analysis of glycolysis lactylated proteins. *G*, Network interaction diagram of lactylated proteins enriched in glycolysis.

The biological functions of 220 lactylated proteins were further analyzed using Gene Ontology (GO) functional annotation and Kyoto Encyclopedia of Genes and Genomes (KEGG) pathway enrichment analysis. In the GO functional annotation, cellular component analysis showed that sperm lactylated proteins were enriched in 9 + 2 motile cilium and sperm flagellum (Fig. 4*A*). Molecular function analysis showed enrichment in cytoskeletal motor activity (Fig. 4*B*). And biological process analysis showed enrichment in fertilization, ATP metabolic process, and sperm motility (Fig. 4*C*). KEGG pathway enrichment results showed that sperm lactylated proteins were enriched in the glycolytic pathway (Fig. 4*D*). In the glycolytic process, we found that PGK2 is lactylated at 10 lysines, including K6, K11, K31, K41, K141, K192, K220, K272, K322, and K353 (Fig. 4*E*). PGK2 is a testis-specific expressed glycolytic enzyme key for glycolysis and ATP generation (Fig. 4, *F* and *G*). Taken together, the lactylation of PGK2 might regulate the generation of ATP and the motility of sperm.

### PGK2 Lactylation Affects Sperm Motility in AZS by Regulating Glycolytic Process

**Fig. 5:**
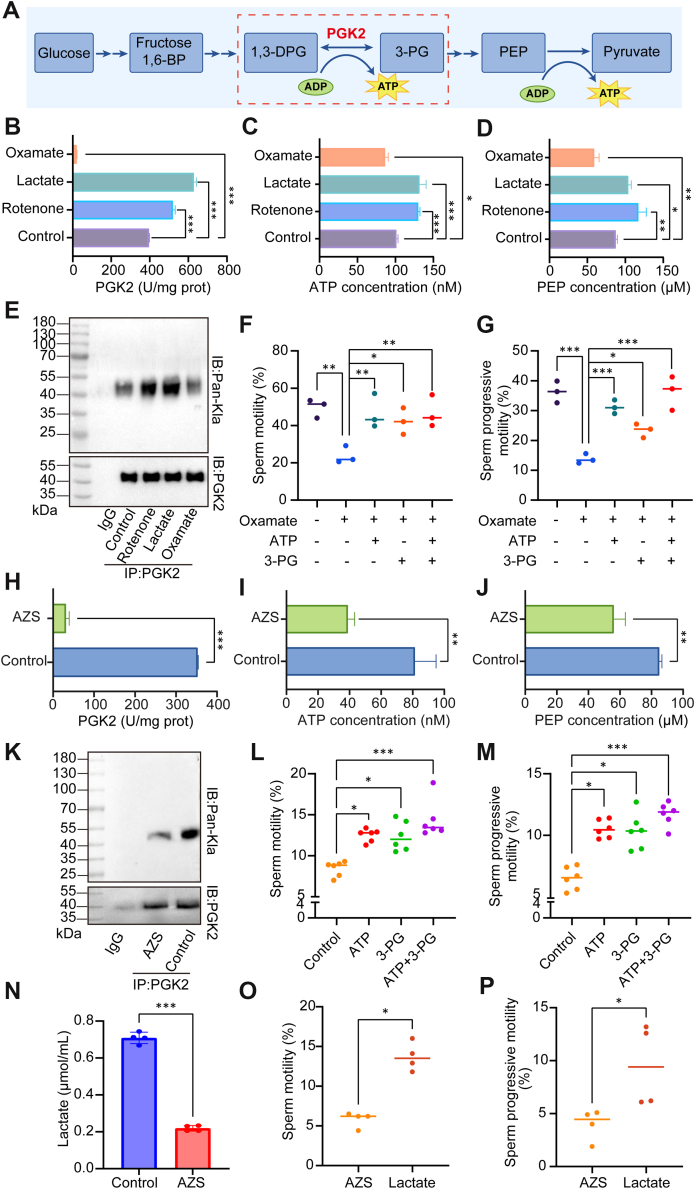
**PGK2 lactylation regulates sperm motility by affecting its enzyme activity.***A*, schematic diagram of PGK2 regulation of glycolysis. *B*, PGK2 enzyme activity, (*C* and *D*) downstream ATP and PEP content in sperm were measured after addition of exogenous lactate, rotenone and oxamate. *E*, the level of PGK2 lactylation was detected by IP experiments. *F* and *G*, the effect of oxamate on sperm motility after ATP and 3-PG supplementation was evaluated. *H*, PGK2 enzyme activity and (*I* and *J*) its downstream ATP and PEP content were measured in sperm of AZS. *K*, IP experiment was used to verify the PGK2 lactylation in the sperm of AZS. *L* and *M*, sperm motility by CASA after supplementation of ATP and/or 3-PG into sperm culture medium. *N*, the level of lactate was detected in AZS sperm. *O* and *P*, AZS sperm motility was evaluated after supplementation with exogenous lactate. All values are mean ± SD, ∗*p* < 0.05, ∗∗∗*p* < 0.001. All values are mean ± SD, ∗*p* < 0.05, ∗∗*p* < 0.01, ∗∗∗*p* < 0.001.

As a key enzyme in the glycolytic pathway, PGK2 catalyzes the conversion of 1,3-bisphosphoglycerate and ADP to 3-phosphoglycerate (3-PG) and ATP (24), which is the first step in ATP production during glycolysis process (Fig. 5*A*). To investigate the impact of PGK2 lactylation on the glycolytic pathway in sperm, rotenone, sodium lactate, and oxamate were individually introduced into normal semen samples. After assaying the enzyme activity of PGK2, it was found that both rotenone and sodium lactate increased the enzyme activity of PGK2, whereas oxamate significantly inhibited the enzyme activity of PGK2 (Fig. 5*B*). Subsequent analyses of PGK2 downstream products indicated that ATP and PEP concentrations were elevated following treatment with rotenone and sodium lactate, but diminished with oxamate exposure (Fig. 5, *C* and *D*). We further analyzed the lactylation levels of PGK2 by immunoprecipitation of PGK2 followed by Western blot with pan-Kla antibody. The results demonstrated that both rotenone and sodium lactate significantly increased PGK2 lactylation levels, whereas oxamate led to a reduction in lactylation levels (Fig. 5*E*). Importantly, the inhibitory effect of oxamate on sperm motility was effectively counteracted by the addition of exogenous ATP and 3-PG (Fig. 5, *F* and *G*).

In clinical sample analyses, PGK2 enzyme activity in AZS sperm was much lower than in normal controls (Fig. 5*H*), and this was accompanied by a significant reduction in the levels of ATP and PEP in seminal plasma (Fig. 5, *I* and *J*). It was also observed that PGK2 lactylation levels in AZS sperm were downregulated (Fig. 5*K*). Sperm motility in AZS was restored through exogenous supplementation with both ATP and 3-PG, and the combination of the two significantly improved sperm motility (Fig. 5, *L* and *M*). In addition, the content of lactate in AZS seminal plasma was notably decreased (Fig. 5*N*), whereas exogenous supplementation with sodium lactate improved sperm motility in AZS (Fig. 5, *O* and *P*), suggesting that lactylation abnormality is important for the defective sperm motility in AZS.

### Lactylation of PGK2 (K220) Affects Glycolysis by Regulating its Enzyme Activity

**Fig. 6:**
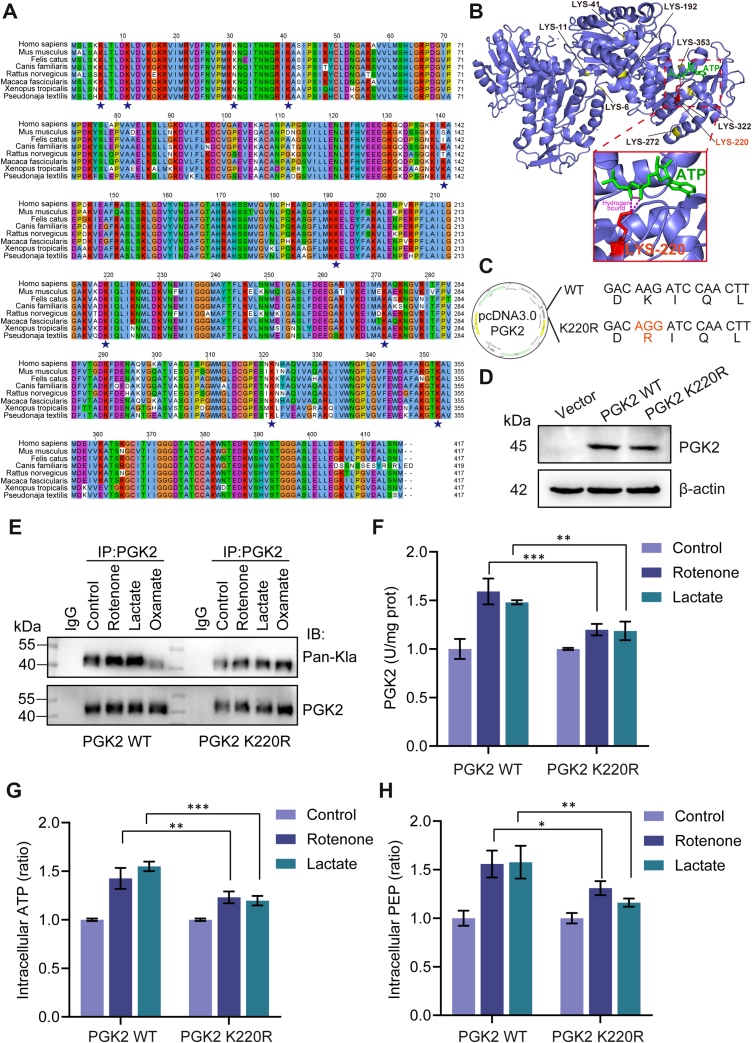
**The lactylation at the K220 site of PGK2 regulates glycolysis by influencing its enzyme activity.***A*, multiple sequence alignment of PGK2 proteins from different species. *B*, schematic structure of PGK2 protein bound to ATP. *C*, Schematic of the point mutation that prevents (K220R) PGK2 220 lactylation. *D*, the transfection efficiency of WT or mutant K220 in 293T cells was verified by Western blot. *E*, the levels of PGK2 lactylation were detected in 293T cells expressing WT or mutated PGK2. *F*, PGK2 enzyme activity and (*G* and *H*) intracellular ATP and PEP were measured in 293T cells transfected with PGK2 WT and PGK2 K220R. All values are mean ± SD, ∗*p* < 0.05, ∗∗*p* < 0.01, ∗∗∗*p* < 0.001.

Sperm lactylation proteomic analysis revealed the presence of 10 lactylation sites (K6, K11, K31, K41, K141, K192, K220, K272, K322, and K353) in the PGK2 protein. Protein sequence conservation analysis across various species demonstrated that PGK2 is highly conserved, including humans, mice, cats, dogs, rats, monkeys and snakes (Fig. 6*A*). Sawyer *et al*. characterized the structure of PGK2 and ATP complex, and found that K220 forms two hydrogen bonds with ATP (25) (Fig. 6*B*). To elucidate the functional significance of K220, a site-specific mutagenesis approach was employed to substitute lysine (K) with arginine (R) at the K220 site (Fig. 6, *C* and *D*). The results demonstrated that the K220R mutation not only reduced the level of PGK2 lactylation (Fig. 6, *E* and *F*) but also effectively reduced the generation of ATP and PEP, two downstream products of PGK2 in the glycolytic pathway (Fig. 6, *G* and *H*). These results suggest that lactylation at K220 site of PGK2 plays a regulatory role in ATP production, thereby affecting sperm motility.

## Discussion

Our analysis of protein Kla showed its important regulatory roles in human sperm motility. Proteomic analysis of human sperm revealed 220 lactylated proteins, enriched in the glycolysis pathway and sperm motility. The level of sperm protein lactylation was down-regulated in patients with AZS. The K220 of PGK2, a protein with abnormally down-regulated lactylation level in AZS, regulated the enzyme activity and the generation of PEP and ATP. The sperm motility defect can be rescued by supplementing downstream products of PGK2 and lactate treatment. Aberrant protein lactylation is an important factor of AZS.

We found that protein lactylation was distributed in the sperm flagellum, acrosome and nucleus. The flagellum is the power device for sperm motility. And the level of protein lactylation was downregulated in AZS sperm. AZS, characterized by diminished sperm motility, is widely recognized as a major contributor to male infertility (2). However, its underlying pathogenesis remains poorly understood. Protein phosphorylation has been demonstrated to regulate sperm motility. The protein kinases PI3K and AKAP3 were involved in phosphoric acid regulation of human sperm motility (26). Tyrosine phosphorylation is an important marker of sperm motility (27), and its decreased phosphorylation level has also been reported to be closely related to AZS (6). Our treatment of human sperm with various interventions in lactylation experiments demonstrated that lactylation is an important regulatory mechanism of sperm motility and proposed a new regulatory mechanism of sperm motility.

This study further identified 220 lactylated proteins in normal sperm through Kla proteomic analysis. Bioinformatics results revealed that the sperm lactylated proteins were enriched in the glycolytic pathway. This finding is highly consistent with the characteristics of sperm energy metabolism, where mature sperm rely predominantly on glycolysis for rapid energy supply due to the absence of mitochondrial DNA and highly condensed chromatin (28). As the end product of glycolysis, the concentration gradient of lactate may play a role by affecting the pH value of the microenvironment or directly participating in protein lactylation. The results of lactylation proteomics showed that a variety of key enzymes in the glycolytic pathway, including PKM, PDHA, PGK2, and GAPDH, were modified by lactylation, indicating that the glycolytic pathway was regulated by lactylation.

PGK2 is a pivotal enzyme within the glycolytic pathway, facilitating the conversion of 1,3-diphosphoglycerate to 3-phosphoglycerate (3-PG) while concurrently generating ATP (24). Our research demonstrated a positive correlation between ATP production and the lactylation level of PGK2. Notably, both PGK2 lactylation and its enzyme activity was significantly diminished in AZS sperm. The enzyme activity of PGK2 has a direct impact on glycolytic flux. In male mice deficient in PGK2, sperm motility and ATP levels were significantly reduced (24). The expression of PGK2 is also decreased in sperm of older men and young AZS patients (29). Furthermore, we found that the motility of AZS sperm was restored upon supplementation with exogenous ATP and 3-PG. The decreased lactylation level of PGK2 may cause impaired sperm motility by reducing enzyme activity in AZS sperm. Among the 10 lactylation sites identified through LC-MS/MS analysis, the crystal structure analysis of PGK2 showed that the lysine of PGK2 K220 was linked to ATP with two hydrophobic bonds. We mutated K220 and confirmed that PGK2 (K220) lactylation is important for its enzyme activity and ATP production. Luo *et al*. previously reported that PGK2 K220 site can also undergo acetylation (30), and there may be potential cross-talk between the two modifications at PGK2 K220, which is worthy of further investigation.

In conclusion, our study found complex protein lactylation in human sperm, observed dysregulation of protein lactylation in AZS sperm, and proposes the important roles of protein Kla in the regulation of sperm motility. The defects of lactylation are important pathogenic mechanisms in AZS. These findings expand the understanding of lactylation of sperm proteins and provide a theoretical basis for precise diagnosis and treatment of male infertility. Future studies are needed to elucidate the enzymatic regulatory network of lactylation and resolve the pathogenesis of AZS.

## Data Availability

The mass spectrometry proteomics data have been deposited to the ProteomeXchange Consortium via the PRIDE partner (https://www.ebi.ac.uk/pride/archive) repository with the dataset identifier PXD064912 and could be accessed via a reviewer account (Username: reviewer_pxd064912@ebi.ac.uk, password: bOoMzTGLketX).

## Supplemental data

This article contains supplemental data.

## Conflict of Interest

The authors declare that they do not have any conflicts of interest with the content of this article.

## References

[1] Zhu C., Yan L., He C., Wang Y., Wu J., Chen L. (2022). Incidence and risk factors of infertility among couples who desire a first and second child in Shanghai, China: a facility-based prospective cohort study. Reprod. Health.

[2] Eisenberg M.L., Esteves S.C., Lamb D.J., Hotaling J.M., Giwercman A., Hwang K. (2023). Male infertility. Nat. Rev. Dis. Primers.

[3] Cooper T.G., Noonan E., von Eckardstein S., Auger J., Baker H.W., Behre H.M. (2010). World Health Organization reference values for human semen characteristics. Hum. Reprod. Update.

[4] Curi S.M., Ariagno J.I., Chenlo P.H., Mendeluk G.R., Pugliese M.N., Sardi Segovia L.M. (2003). Asthenozoospermia: analysis of a large population. Arch. Androl..

[5] Wu Y., Yuan Y., Chen L., Wang M., Yang Y., Wang Y. (2019). Quantitative proteomic analysis of human seminal plasma from normozoospermic and asthenozoospermic individuals. Biomed. Res. Int..

[6] Tu C., Wang W., Hu T., Lu G., Lin G., Tan Y.Q. (2020). Genetic underpinnings of asthenozoospermia. Best Pract. Res. Clin. Endocrinol. Metab..

[7] Amoako A.A., Marczylo T.H., Elson J., Taylor A.H., Willets J.M., Konje J.C. (2014). Relationship between seminal plasma levels of anandamide congeners palmitoylethanolamide and oleoylethanolamide and semen quality. Fertil. Steril.

[8] Zhu C.H., Wei Y., Chen F., Li F., Zhang S.M., Dong N.J. (2023). Investigation on the mechanisms of human sperm DNA damage based on the proteomics analysis by SWATH-MS. Clin. Proteomics.

[9] Amann R.P., Schanbacher B.D. (1983). Physiology of male reproduction. J. Anim. Sci..

[10] Baker M.A., Witherdin R., Hetherington L., Cunningham-Smith K., Aitken R.J. (2005). Identification of post-translational modifications that occur during sperm maturation using difference in two-dimensional gel electrophoresis. Proteomics.

[11] Brohi R.D., Huo L.J. (2017). Posttranslational modifications in spermatozoa and effects on Male fertility and sperm viability. OMICS.

[12] Pieroni L., Iavarone F., Olianas A., Greco V., Desiderio C., Martelli C. (2020). Enrichments of post-translational modifications in proteomic studies. J. Sep. Sci..

[13] Guo Y., Jiang W., Yu W., Niu X., Liu F., Zhou T. (2019). Proteomics analysis of asthenozoospermia and identification of glucose-6-phosphate isomerase as an important enzyme for sperm motility. J. Proteomics.

[14] Zhang D., Tang Z., Huang H., Zhou G., Cui C., Weng Y. (2019). Metabolic regulation of gene expression by histone lactylation. Nature.

[15] Izzo L.T., Wellen K.E. (2019). Histone lactylation links metabolism and gene regulation. Nature.

[16] Notarangelo G., Haigis M.C. (2019). Sweet temptation: from sugar metabolism to gene regulation. Immunity.

[17] Dong H., Zhang J., Zhang H., Han Y., Lu C., Chen C. (2022). YiaC and CobB regulate lysine lactylation in Escherichia coli. Nat. Commun..

[18] Ding T., Yang Y.H., Wang Q.C., Wu Y., Han R., Zhang X.T. (2024). Global profiling of protein lactylation in Caenorhabditis elegans. Proteomics.

[19] Wan N., Wang N., Yu S., Zhang H., Tang S., Wang D. (2022). Cyclic immonium ion of lactyllysine reveals widespread lactylation in the human proteome. Nat. Methods.

[20] Wang J., Wang Z., Wang Q., Li X., Guo Y. (2024). Ubiquitous protein lactylation in health and diseases. Cell Mol. Biol. Lett..

[21] Li H., Sun L., Gao P., Hu H. (2024). Lactylation in cancer: current understanding and challenges. Cancer Cell.

[22] Li F., Si W., Xia L., Yin D., Wei T., Tao M. (2024). Positive feedback regulation between glycolysis and histone lactylation drives oncogenesis in pancreatic ductal adenocarcinoma. Mol. Cancer.

[23] Wang X., Ying T., Yuan J., Wang Y., Su X., Chen S. (2023). BRAFV600E restructures cellular lactylation to promote anaplastic thyroid cancer proliferation. Endocr. Relat. Cancer.

[24] Danshina P.V., Geyer C.B., Dai Q., Goulding E.H., Willis W.D., Kitto G.B. (2010). Phosphoglycerate kinase 2 (PGK2) is essential for sperm function and male fertility in mice. Biol. Reprod..

[25] Sawyer G.M., Monzingo A.F., Poteet E.C., O'Brien D.A., Robertus J.D. (2008). X-ray analysis of phosphoglycerate kinase 2, a sperm-specific isoform from Mus musculus. Proteins.

[26] Luconi M., Baldi E. (2003). How do sperm swim? Molecular mechanisms underlying sperm motility. Cell Mol. Biol. (Noisy-le-grand).

[27] Visconti P.E., Bailey J.L., Moore G.D., Pan D., Olds-Clarke P., Kopf G.S. (1995). Capacitation of mouse spermatozoa. I. Correlation between the capacitation state and protein tyrosine phosphorylation. Development.

[28] du Plessis S.S., Agarwal A., Mohanty G., van der Linde M. (2015). Oxidative phosphorylation versus glycolysis: what fuel do spermatozoa use?. Asian J. Androl..

[29] Liu X.X., Zhang H., Shen X.F., Liu F.J., Liu J., Wang W.J. (2016). Characteristics of testis-specific phosphoglycerate kinase 2 and its association with human sperm quality. Hum. Reprod..

[30] Tian Y., Wang H., Pan T., Hu X., Ding J., Chen Y. (2024). Global proteomic analyses of lysine acetylation, malonylation, succinylation, and crotonylation in human sperm reveal their involvement in male fertility. J. Proteomics.

